# Single‐Scan Selective Excitation of Individual NMR Signals in Overlapping Multiplets

**DOI:** 10.1002/anie.202011642

**Published:** 2020-11-09

**Authors:** Peter Kiraly, Nicolas Kern, Mateusz P. Plesniak, Mathias Nilsson, David J. Procter, Gareth A. Morris, Ralph W. Adams

**Affiliations:** ^1^ Department of Chemistry University of Manchester Oxford Road Manchester M13 9PL UK

**Keywords:** natural products, NMR spectroscopy, nuclear Overhauser effect, selectivity, structure elucidation

## Abstract

2D NMR is an immensely powerful structural tool but it is time‐consuming. Targeting individual chemical groups by selective excitation in a 1D experiment can give the information required far more quickly. A major problem, however, is that proton NMR spectra are often extensively overlapped, so that in practice only a minority of sites can be selectively excited. Here we overcome that problem using a fast, single‐scan method that allows selective excitation of the signals of a single proton multiplet even where it is severely overlapped by other multiplets. The advantages of the method are illustrated in a selective 1D NOESY experiment, the most efficient way to determine relative configuration unambiguously by NMR. The new approach presented here has the potential to broaden significantly the applicability of selective excitation and unlock its real potential for many other experiments.

## Graphical abstract

Selective excitation of overlapped multiplets in a single scan broadens the applicability of 1D selective NMR experiments.

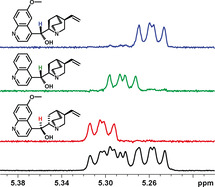

NMR spectroscopy is the chemist's primary tool for determining the structures and dynamics of organic molecules. It is ubiquitous in chemistry laboratories, and critical at all points in chemical synthesis, from the monitoring and confirmation of success of reactions to the determination of chemical structure and configuration. The determination of structure and configuration by NMR spectroscopy is heavily dependent on 2D NMR experiments. In these experiments, nuclei close in space or scalar coupled through chemical bonds give signals which identify proximity or connectivity, allowing structure, including relative configuration, to be established.

The process of structure determination rarely necessitates the analysis of the interactions of all protons, as yielded by time‐consuming 2D experiments. Instead, selective excitation of individual signals allows their connectivities and interactions with other nuclei to be probed.^[1]^ Such selective 1D experiments often have the capability to provide all the information required for structure elucidation in a fraction, as low as a percent or so, of the 2D experiment time.^[2]^ They use selective pulses to excite a specific signal of interest only. Suitable selective pulses for such experiments exist for a wide range of applications[^1^, ^3^] but have a common limitation: where multiplet structure in proton NMR spectra causes overlap between signals, a single signal cannot be excited without perturbing others. Here we report a novel approach, a gradient‐enhanced multiplet‐selective targeted‐observation NMR experiment (GEMSTONE), that gives unprecedented selectivity and for the first time allows a single multiplet to be extracted from an overlapped region of many proton signals in a single scan.

The overlap problem is illustrated in Figure 1 for the steroid 17β‐estradiol. The proton NMR spectrum between 1.7 and 1.9 ppm contains overlapping multiplets from protons 7β, 12β and 16α which are in three different rings of the structure. Conventional selective excitation (Figure 1 a) selects all three protons simultaneously, making the NOEs observed in a 1D NOESY experiment ambiguous (black arrows in Figure 1), and useless for structure elucidation. In contrast, the different NOEs of the three multiplets can be observed individually using GEMSTONE (Figure 1 b–d, gray arrows). The selectivity achieved allows unambiguous interpretation of the NOESY spectra and assignment of the stereochemistry. For example, ambiguity in the assignment of proton‐12 (α or β) can be resolved by identifying an NOE with either of protons 18 or 17, which are respectively above and below the plane of the D‐ring of the sterane structure. Each selective 1D spectrum provides the same information as a single trace extracted from a 2D spectrum, but takes only a fraction of the time to acquire.

**Figure 1:**
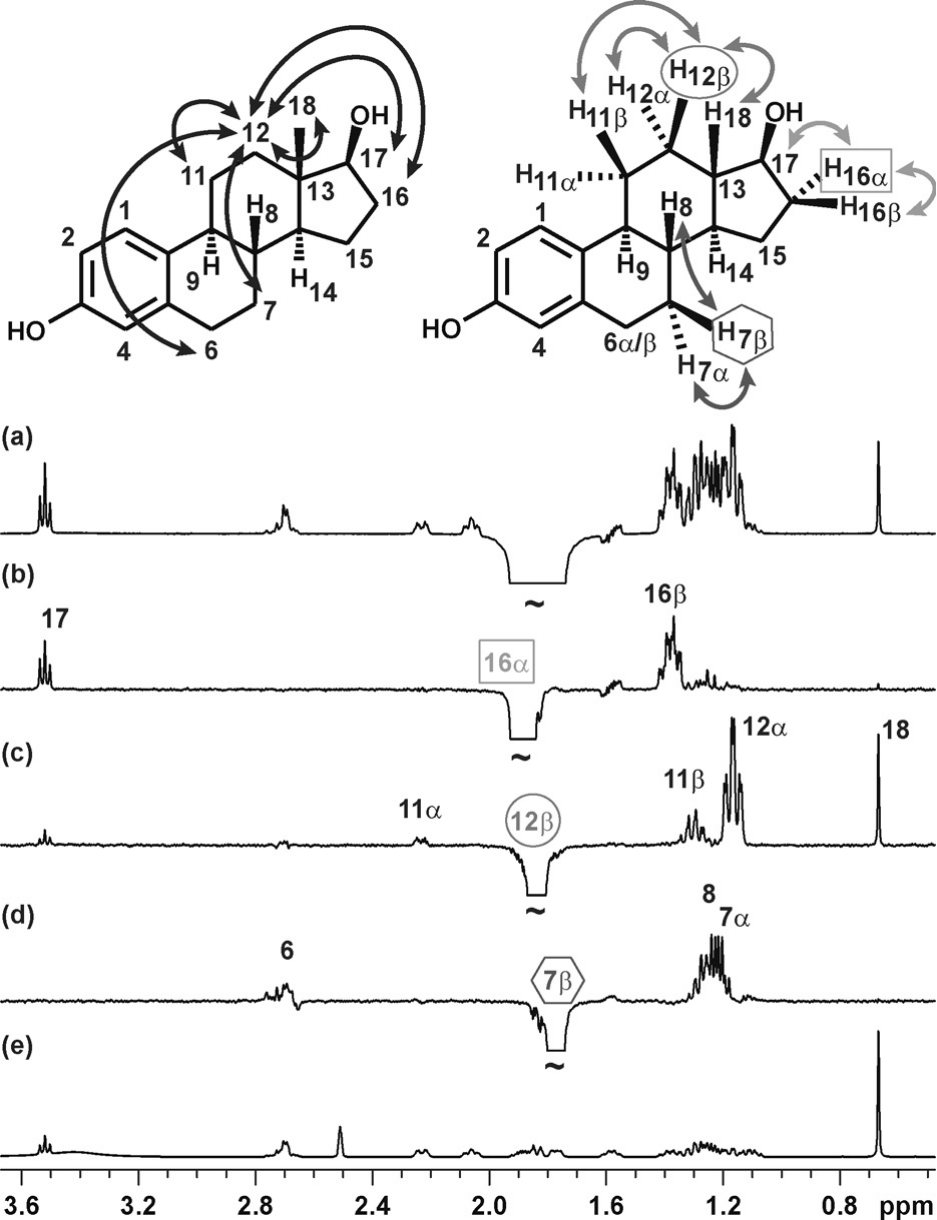
a) Conventional selective 1D NOESY, b)–d) GEMSTONE NOESY, and e) proton NMR spectra of estradiol in [D_6_]DMSO. Unambiguous interpretation requires the unique selectivity provided by the GEMSTONE approach. The experiment times for the NOE experiments (a–d) were 12 minutes; 128 scans were collected.

Several approaches have been developed previously that can excite individual overlapping signals, but they all suffer from complications and for routine use all require long experiment times. They include two‐step experiments that excite a well‐resolved signal and then use targeted transfer of magnetization to the proton of interest,[^4^, ^5^, ^6^, ^7^] and multi‐dimensional approaches such as HSQC‐NOESY,^[8]^ which exploits the high resolution of the ^13^C spectrum. A more direct way to selectively observe a single site from within multiple overlapping signals is the chemical shift selective filter (CSSF),[^9^, ^10^, ^11^, ^12^] which is the starting point for the method presented here. In a CSSF experiment a variable evolution period gives all off‐resonance signals an offset‐dependent phase modulation. Summing a series of spectra with different evolution times retains the chosen on‐resonance signal but attenuates all others.

Despite its excellent selectivity, the CSSF is not in common use, which is due to its long experiment duration and complexity of configuration. Related approaches using semi‐selective excitation in homonuclear 2D experiments have been used as an alternative,[^13^, ^14^] but it is much commoner to fall back on time‐consuming 2D experiments. These can represent a very inefficient use of precious NMR instrument time. GEMSTONE in contrast delivers a fast, generally applicable solution to the problem of selectively exciting multiplets. The key advantages over the CSSF are that it uses single‐scan acquisition (minimizing experiment time) and avoids the need for tedious parameter optimization (enabling straightforward implementation for automation). Section 2 of the Supporting Information gives a critical comparison of the two methods.

Pulse sequences for basic selective excitation of a single multiplet and for 1D selective NOESY measurement are shown in Figure 2. The GEMSTONE approach uses a novel pulse sequence element for continuous spatial encoding of the chemical shift. A spatially multiplexed chemical shift selective filter is produced by the two adiabatic pulses (*τ*_p_) applied in the presence of magnetic field gradients (*G*_1_) flanking a conventional (band‐)selective refocusing pulse. The spins refocused by the latter acquire a phase shift that depends both on their vertical position in the sample and on their chemical shift. The adiabatic pulses affect different resonance offsets at different times, so only spins with their chemical shift exactly on resonance end up with the same precession phase throughout the sample. All others acquire a chemical shift‐dependent phase shift that varies with position, with the result that their signals cancel when a free induction decay (FID) is recorded. There is an analogy here with a different use of spatial multiplexing, in UF (Ultrafast) nD NMR experiments,[^15^, ^16^] but the latter use technically very challenging signal acquisition strategies derived from magnetic resonance imaging rather than conventional FID acquisition. A further difference between the spatial encoding in UF NMR and that in GEMSTONE is that in the latter the (band‐)selective 180° pulse inverts the spins of interest, but not any spins to which those are coupled, thus suppressing the undesirable modulation caused by *J* coupling. The high sensitivity penalty typical of UF NMR, incurred by the high bandwidth and low effective acquisition time used, does not apply to GEMSTONE, where conventional acquisition is employed. A sensitivity comparison between conventional selective excitation and GEMSTONE, used with comparable bandwidths to excite a well‐resolved signal, showed better signal‐to‐noise ratio for GEMSTONE (Supporting Information, Figure S19).

**Figure 2:**
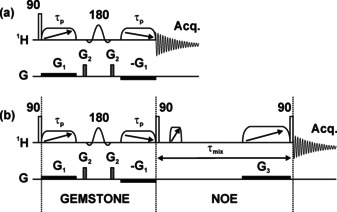
Pulse sequences for a) the basic gradient‐enhanced multiplet‐selective targeted‐observation NMR experiment (GEMSTONE) and b) 1D GEMSTONE NOESY. Open rectangles, diagonal arrow crossed elements, and sinc shapes represent excitation, adiabatic, and band‐selective pulses, respectively. The effective bandwidth of the adiabatic pulses is matched to the applied gradient *G*_1_. The time for chemical shift evolution is spatially encoded to cover the range −*τ*_p_ to +*τ*_p_.

The GEMSTONE element effectively performs a continuous range of different experiments across the sample, averaging a continuum of *t*_1_ evolution periods in a single scan. A further analogy is with zero‐quantum suppression (ZQS) in TOCSY and NOESY experiments. Early methods averaged multiple different acquisitions, but were superseded by Keeler's single‐scan ZQS element.^[17]^ In both cases the key is spatial multiplexing, using the different parts of the sample to achieve in a single experiment the effect of averaging multiple experiments under different conditions.

The selectivity of GEMSTONE is limited by the sum (2*τ*_p_) of the durations of the two adiabatic pulses, reduced slightly by the smoothing of their amplitudes at the edges of the pulses. Just as the maximum evolution time *t*_1_^max^ determines the *F*_1_ resolution of a 2D experiment, so 2*τ*_p_ determines the limiting selectivity of GEMSTONE, giving a sinc dependence of signal amplitude on resonance offset with excitation crossing zero at approximately ±1/*τ*_p_ Hz. The sinc GEMSTONE excitation profile is sufficient for most purposes, but the profile could if required be tailored by changing the time dependence of the frequency sweep and the field gradient pulse (*G*_1_). Relaxation during 2*τ*_p_ causes a sensitivity penalty, as in constant‐time 2D experiments,^[18]^ but does not affect the selectivity. Experiments, and numerical simulations using the Spinach program,^[19]^ confirm the predicted selectivity of the GEMSTONE method (Supporting Information, Figures S2, S3).

The power of GEMSTONE for disentangling overlapped proton multiplets is illustrated by the ^1^H NMR spectrum (Figure 3 a) of the diastereomeric carbocyclic product **1** of an asymmetric synthesis.^[20]^ Α small region of this spectrum, containing a doublet and two methyl triplets, is shown in Figure 3 b; it also contains the methyl signal (17 m) of a minor product. The less shielded methyl (17) is particularly important for structure verification because of its proximity to the chiral center (5) for which configuration needs to be determined. Classical selective excitation of multiplet 17 is impossible because of overlap, but GEMSTONE distinguishes between the signals, allowing selective excitation of methyl 17 and subsequent clean analysis of selective 1D NOESY experiments.

**Figure 3:**
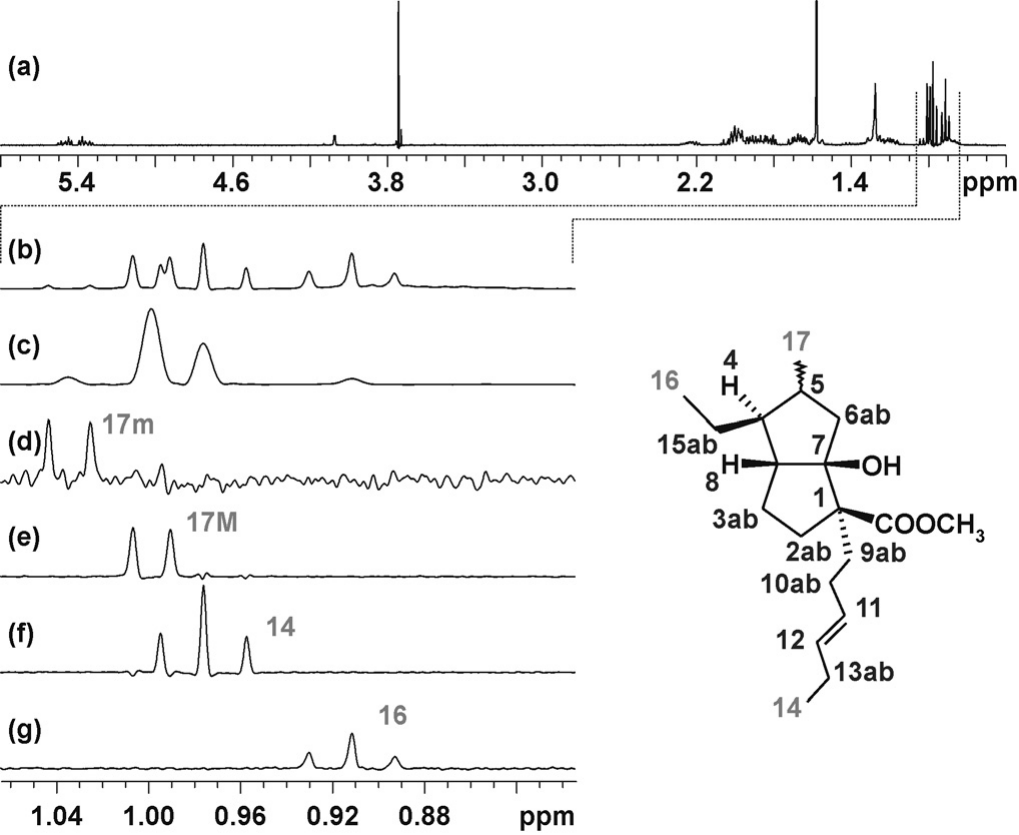
a) ^1^H NMR spectrum of a diastereomeric mixture at C5, carbocycle **1**. b)–e) Expansions showing overlapping protons using b) conventional ^1^H experiment, c) band‐selective pure shift spectrum, d)–g) selective excitation of each of the multiplet using GEMSTONE. The transmitter offset for the GEMSTONE experiments were peak picked from the pure shift NMR spectrum. The GEMSTONE experiments (d–g) used 8 scans, to provide sufficient sensitivity for detection of the minor component.

As a first step a band‐selective (or if multiple regions are needed then a broadband, for example, Zangger–Sterk) pure shift NMR^[21]^ experiment is carried out in which multiplet structures are collapsed and a single peak is observed for each chemically non‐equivalent proton (Figure 3 c). The pure shift experiment is used only for peak picking, so data can be acquired quickly with low resolution. The sample used is particularly challenging because of the presence of the minor component, so the recently introduced SAPPHIRE method^[22]^ was used to get the highest spectral purity. The pure shift NMR experiment took only 3 min (in a modest 400 MHz spectrometer with room temperature probe) for this sensitivity‐limited example in which the concentration of the main component is ca. 40 μM. A further time saving could have been achieved if needed by semi‐real‐time acquisition.^[23]^ The selectivity of GEMSTONE enables clean extraction of all signals, including that of the minor component (see Figures 3 d–g).

GEMSTONE is fully compatible with automated spectrometer use, enhancing its potential impact. The only user input required is to define the overlapped region of the spectrum from which multiplets need to be extracted. The pure shift spectrum can be processed with automatic peak picking, and the selected frequencies used to set the transmitter offset for a set of GEMSTONE experiments.

The selective extraction of a single multiplet from underneath other signals is useful for extracting spectral components, but the greater benefits of GEMSTONE are realized in experiments such as selective 1D NOESY. The region around 1.2 ppm in the ^1^H NMR spectrum of carbocycle **1** shows severe overlap of three multiplets and illustrates the use of GEMSTONE for ultra‐selective observation of key NOEs (Figure 4). Normal selective excitation of individual protons in this spectral region is not possible, which renders the classic 1D NOESY experiment unsuitable. With the additional selectivity of GEMSTONE, independent excitation of each of the overlapped multiplets is possible and their NOEs can be analyzed independently. No useful information can be extracted from the severely overlapped signals at 1.65 ppm in the classic 1D NOESY spectrum of Figure 4 a. In contrast, the GEMSTONE spectra 4b‐d provide both the trivial NOE contacts 2b‐3b and 15a‐15b and the stereospecific contacts 6b‐9b and 6b‐2b that allow unambiguous assignment of protons 6a and 6b. Further selective spectra for carbocycle **1** are provided in the Supplementary Information, along with other illustrative examples including selective excitation of strongly coupled multiplets in an analogue of carbocycle **1**, and of signals from the highly overlapped spectrum of a mixture of cinchona alkaloids.

**Figure 4:**
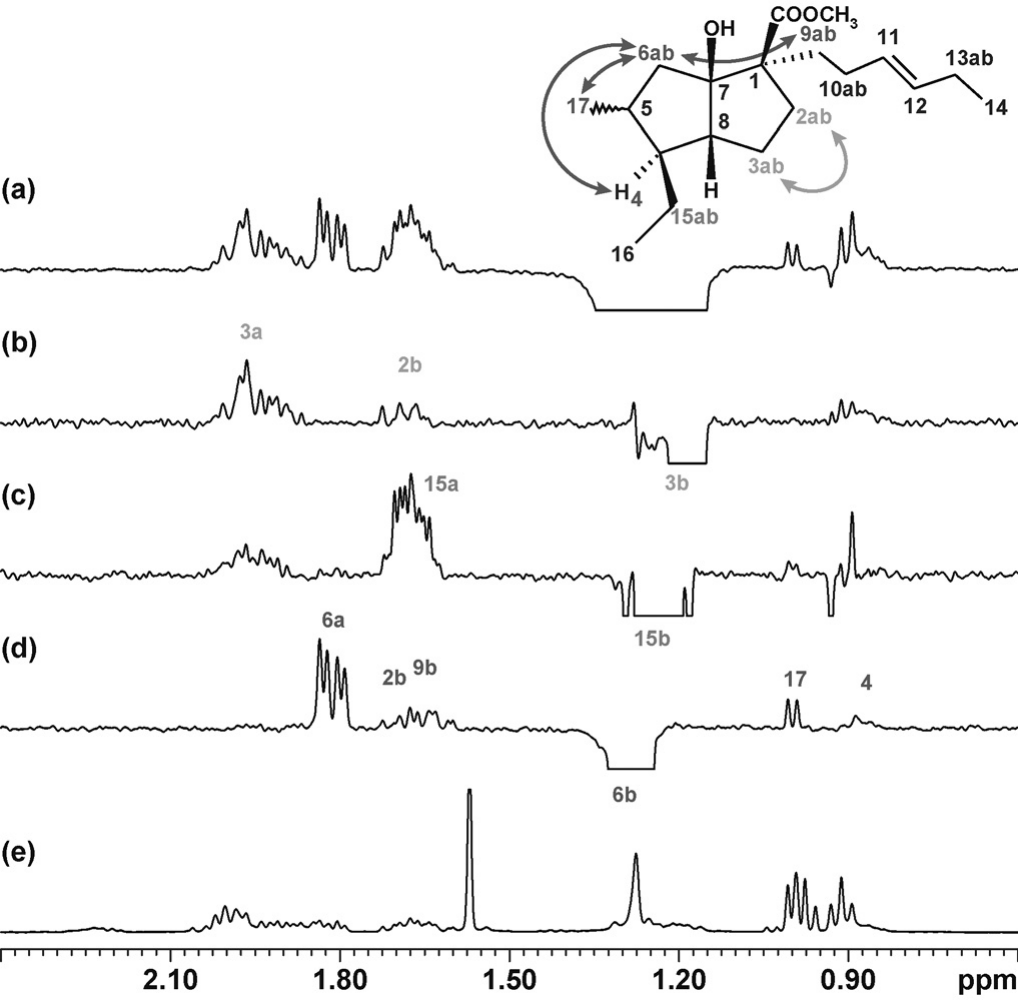
a) Conventional selective 1D NOESY using a 100 ms REBURP pulse, b)–d) GEMSTONE NOESY experiments using 100 ms effective *t*_1_^max^, and e) ^1^H NMR spectra of a diastereomeric mixture (carbocycle **1**). Unambiguous interpretation of key NOEs from severe overlap of three protons (6b, 15b, and 3b) requires the unique selectivity provided by GEMSTONE. The NOE experiments (a–d) used 4096 scans because of the very low concentration (ca. 40 μM) of the main component of this mixture.

An NMR pulse sequence element, GEMSTONE, has been introduced and demonstrated that exploits simultaneous field gradients and swept‐frequency pulses to select individual NMR signals in a spectrum irrespective of multiplet structure. The sequence gives unprecedented signal selectivity and works in a single scan, simultaneously performing a continuous range of experiments across the sample to remove unwanted, off‐resonance, signals. The pulse sequence element can be conveniently implemented in any selective excitation experiment without increasing the minimum experiment time required. Applications of the sequence to 1D NOESY measurement in complex spectra have been demonstrated. Selective 1D TOCSY, COSY, ROESY, and relaxation experiments are all compatible with GEMSTONE, and will be the subject of further research. The increased selectivity of the pulse sequence element will greatly extend the applicability of selective 1D methods, replacing time‐consuming homonuclear 2D experiments. Other potential applications include measuring the diffusion coefficient of a single component in a complex mixture, and simplification of reaction monitoring by NMR spectroscopy.

## Conflict of interest

The authors declare no conflict of interest.

## Supporting information

As a service to our authors and readers, this journal provides supporting information supplied by the authors. Such materials are peer reviewed and may be re‐organized for online delivery, but are not copy‐edited or typeset. Technical support issues arising from supporting information (other than missing files) should be addressed to the authors.

Supplementary
