# Supplementary material for: Single‐Scan Selective Excitation of Individual NMR Signals in Overlapping Multiplets
Source: Angew Chem Int Ed Engl. 2020 Nov 9;60(2):666–9. doi: 10.1002/anie.202011642 (PMC7839668; doi:10.1002/anie.202011642)
Supplement: Supplementary file 1 — Supplementary [file ANIE-60-666-s001.pdf]

Angewandte Chemie
Eine Zeitschrift der Gesellschaft Deutscher Chemiker

Supporting Information

Single-Scan Selective Excitation of Individual NMR Signals in Overlapping Multiplets

Peter Kiraly, Nicolas Kern, Mateusz P. Plesniak, Mathias Nilsson, David J. Procter, Gareth A. Morris, and Ralph W. Adams**

anie_202011642_sm_miscellaneous_information.pdf

Author Contributions

P.K. Conceptualization: Equal; Data curation: Lead; Funding acquisition: Supporting; Investigation: Lead; Methodology: Equal; Writing—Original Draft: Lead; Writing—Review & Editing: Equal
N.K. Conceptualization: Supporting; Investigation: Supporting; Resources: Supporting
M.P. Investigation: Supporting; Resources: Supporting
M.N. Conceptualization: Equal; Funding acquisition: Supporting; Supervision: Supporting; Writing—Review & Editing: Supporting
D.P. Funding acquisition: Supporting; Supervision: Supporting; Writing—Review & Editing: Supporting
G.M. Funding acquisition: Lead; Resources: Equal; Supervision: Equal; Validation: Equal; Writing—Review & Editing: Equal
R.A. Conceptualization: Equal; Methodology: Equal; Project administration: Lead; Resources: Lead; Supervision: Equal; Writing—Original Draft: Supporting; Writing—Review & Editing: Equal.

Table of Contents

1. Experimental Procedures ... 1
2. Comparison of CSSF and GEMSTONE ... 2
3. Example: 17β-estradiol ... 5
4. Example: Carbocycle 1 ... 6
5. Example: Carbocycle 2 ... 10
6. Example: mixture of cinchona alkaloids ... 18
7. Author Contributions ... 20

1. Experimental Procedures

NMR experiments were carried out using 400 MHz Bruker AV III (TopSpin 3.2.6), 500 MHz Bruker Avance Neo (TopSpin 4.0.7) spectrometers. An archive containing all of the reported example data, pulse programs and macros is available from our website (https://www.nmr.chemistry.manchester.ac.uk) and DOI: 10.17632/xznbxgfpxx.1. The reader is directed to the data archive for convenient access to experimental parameter details. The pulse program implementations of GEMSTONE are compatible with the standard pulse shaping software packages in Topspin, allowing on-the-fly waveform generation based on user-customisable parameters. The minimum hardware capability needed to perform such experiments is a probe equipped with pulsed field gradients along the z-axis, and a console capable of delivering swept-frequency pulses; (both of these capabilities are standard on almost all modern NMR equipment. Availability of transverse (*xy*) pulsed field gradients is advantageous, reducing the likelihood of accidental signal refocusing, but not essential. The large waveform storage memory provided by the most recent systems (e.g. Bruker Avance NEO) is an advantage as it allows pulse durations to be changed at the discretion of the user; older consoles are more limited as the number of data points that can be used to describe a pulse is smaller. For legacy systems (e.g. Bruker AV) the waveform resolution needs to be adjusted for long adiabatic waveforms in order to avoid exceeding the available memory of the acquisition CPU.

The 17β-estradiol sample concentration was 62 mM in DMSO-d_6. The carbocycle samples contained respectively ca. 40 µM methyl-(1*S*,3a*R*,4*S*,5*R*,6a*R*)-4-ethyl-1-((*E*)-hex-3-en-1-yl)-6a-hydroxy-5-methyloctahydro-pentalene-1-carboxylate (carbocycle **1**), and 4 mM methyl-(1*S*,3a*R*,5*S*,6a*R*)-1-(but-3-en-1-yl)-6a-hydroxy-5-isopropyloctahydropentalene-1-carboxylate (Carbocycle **2**), in $CDCl_3$. Excitation profile tests were carried out with a sealed sample containing 1% v/v H2O in D_2O doped with ca. 0.1 mg/ml GdCl3 (T_1 = 0.2 s for HDO peak at 500 MHz). The cinchona alkaloid mixture contained 35, 29, and 24 mM of quinine, quinidine and cinchonidine in DMSO-d_6.

The pulse sequence diagram for a conventional chemical shift selective filter (CSSF) is shown in Figure S1. This sequence was used as included in the standard Bruker TopSpin library (selcssf). The VT (variable time) chemical shift evolution is not refocused during the t_1/2 periods, and this provides the CSSF filter in the sequence. The parameter setup is complicated, requiring calculations involving the chemical shift differences between the overlapping signals, but when the pulse program is executed it works as depicted with a simple incremented evolution time t_1.

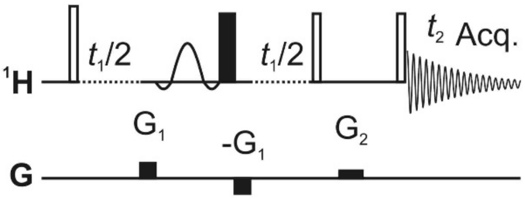

Figure S1. Pulse sequence diagram for a VT-CSSF experiment. Open and filled rectangles indicate 90° and 180° pulses respectively; the shaped form denotes a frequency selective waveform such as Reburp or Rsnob. A series of experiments is acquired with an incremented period t_1 and the resulting fids are added together to produce the CSSF spectrum. VT stands for variable time, reflecting the varying time between excitation and detection, and hence the increasing relaxation loss as t_1 increases. This may distort the excitation profile compared to that for a constant time (CT) approach.

The GEMSTONE pulse sequence of Figure 2 is an inherently constant-time method in which the evolution time of Figure S1 is replaced by a spatial encoding period containing two adiabatic inversion pulses applied under a field gradient. The pulses are of

duration τ_p and their frequencies sweep in opposite directions, from ν – *bw*/2 to ν + *bw*/2 and vice versa, with the frequency range bw matched to the field gradient G_1, sample length L and gyromagnetic ratio γ such that $bw = \gamma G_1 L/(2\pi)$. ν is the frequency of the signal of interest. If $bw > \gamma G_1 L/(2\pi)$ the selectivity is reduced but full sensitivity retained, while if $bw < \gamma G_1 L/(2\pi)$ full selectivity is achieved but sensitivity is reduced, in both cases pro rata. In practice, the adiabatic pulse bandwidth should be slightly more than $\gamma G_1 L/(2\pi)$ to allow for the effects of the pulse shape smoothing. No special calibration is needed, *G* and *L* typically being determined during spectrometer installation. At one end of the sample, spins resonating at frequency $\Delta\nu$ are inverted at either end of the period $2\tau_p$, and at the other they are inverted at its midpoint. The effective phases of the inversion pulses are offset-dependent in an adiabatic swept frequency pulse, so the net result is that spins experience a chemical shift evolution time of $-\tau_p$ at one end of the sample and $+\tau_p$ at the other.

The following typical parameters were used: adiabatic swept frequency pulses were WURST-80 waveforms with τ_p = 100 ms, *bw* = 2.5 kHz, and G_1 = 0.25 G cm^{-1}. The power levels of the WURST-80 pulses were calculated for an adiabaticity factor (Q) of 11, and step size was fixed at 5 μs. No phase cycling is needed for the GEMSTONE sequence; this makes it particularly easy to integrate into phase cycled experiments such as selective 1D NOESY. The band-selective refocusing pulse in GEMSTONE can be shorter, i.e. less selective, than that used in the conventional CSSF approach because its purpose is to refocus homonuclear *J* evolution, not to provide selectivity of excitation. In the CSSF experiment, in contrast, the corresponding pulse is also required to reject the equidistant multiple frequency off-resonance excitations otherwise characteristic of the CSSF method (see Figure S3a below). The selective 180° pulse had a typical excitation bandwidth of 100 Hz, using either RSNOB or RE-BURP waveforms and was flanked by gradient pulses (1 ms, 8-12 G cm^{-1}) to enforce the desired coherence transfer pathway (CTP) without the need for phase cycling. The strengths and durations of the CTP gradient pulses are chosen to avoid accidental refocusing of the effects of G_1. If a triaxial gradient system is available the CTP pulses should be in the *xy* plane, leaving the conventional z-axis for the spatial encoding gradient (G_1). The NOE part of the sequence of Figure 2b is identical to that in a standard 1D NOESY sequence, and typical parameters were used, including an 800 ms mixing time.

2. Comparison of CSSF and GEMSTONE

The GEMSTONE method may be regarded as a single scan analogue of the CSSF experiment.

The most commonly used implementation of the CSSF (Figure S1) consists of an excitation pulse followed by an incremented evolution period t_1 with a semi-selective 180° refocusing pulse at its midpoint, and a z-filter at its end to purge phase signals that are not in phase. The selective 180° pulse serves two purposes, to refocus the effects of couplings to spins that are outside the selective pulse bandwidth, and to limit the range of resonance excited in order to suppress sideband excitation (see below).

The signals that result are amplitude modulated as a function of their offset from resonance $\Delta\nu$, so that when multiple FIDs measured with incremented values of t_1 are averaged, only on resonance signals interfere constructively and others are attenuated. In the limit of a large number of closely-spaced increments in t_1 with a maximum value t_1^{max}, the excitation profile approaches the form of a *sinc* function, $\sin(2\pi\ \Delta\nu\ t_1^{max})/(2\pi\ \Delta\nu)$. In practice it is not practical to approach this limit, and the finite spacing between t_1 values results in sidebands, *sinc* function excitation maxima replicated at multiples of the inverse of the evolution time increment Δt_1, as seen in Figure S3a below. In CSSF the bandwidth of the selective 180° pulse is chosen so as to attenuate these sidebands to an acceptable level. Transverse relaxation during t_1 broadens the excitation spectrum. The averaging of multiple different acquisitions in the CSSF method means that its minimum experiment time is much longer than those for other selective 1D methods; it is essentially a 2D acquisition processed to yield a 1D spectrum. For optimum efficiency the chemical shifts of the overlapping signals need to be known beforehand and used to choose Δt_1 in the current implementation of CSSF in Bruker's TopSpin software.

In the GEMSTONE experiment, in contrast, a continuous range of evolution times from $-\tau_p$ to $+\tau_p$ is sampled in a single scan. The excitation spectrum takes the same form as CSSF, $\sin(2\pi\ \Delta\nu\ t_1^{max})/(2\pi\ \Delta\nu)$, where $t_1^{max} = 2\tau_p$, but without the need for a *z* filter. Because the range of evolution times is continuous, the excitation spectrum contains only a single *sinc* function, with no repetitive sidebands, and there is no need to estimate the chemical shift differences of the overlapping signals beforehand in order to choose a value for Δt_1. The constant sequence duration reduces sensitivity slightly compared to VT CSSF, but means that the excitation spectrum is independent of the transverse relaxation time (similar to CT CSSF). Because there are no repetitive sidebands in GEMSTONE, the bandwidth of the selective 180° pulse is determined only by the need to refocus *J* evolution; in practice this usually means that a shorter pulse, with smaller relaxation losses, is sufficient. A further advantage of GEMSTONE is that signals are selected before data acquisition, not afterwards as in CSSF. This means that it is much more tolerant of high dynamic range samples, allowing weak signals to be cleanly selectively excited in the presence of strong ones, and allowing the full receiver gain to be used.

Figures S3 and S4 show respectively excitation spectra calculated using Spinach simulations and measured experimentally. Parameters in Spinach calculations were set to be close to those used to acquire the experimental data. The same WURST-80 waveforms were used, with 2.5 kHz bandwidth and power level adjusted for an adiabaticity factor (Q) of 11, but a greater step size of 40 μs (slightly adjusted as needed for different durations τ_p) in order to reduce calculation time. Experiments used a 5 μs step size for convenience, as this is an integer submultiple of any common values of pulse duration. The gradient coil size in the simulation was 15 mm, and G_1 was set to 0.67 G cm^{-1}. The small differences between the simulated and experimental GEMSTONE excitation

spectra are attributable in part to the spatial non-uniformity of the magnetic field gradient in the probe used. While the experimental spectra show slightly poorer selectivity close to resonance, further from resonance the suppression of unwanted signals is rather better than in the simulations.

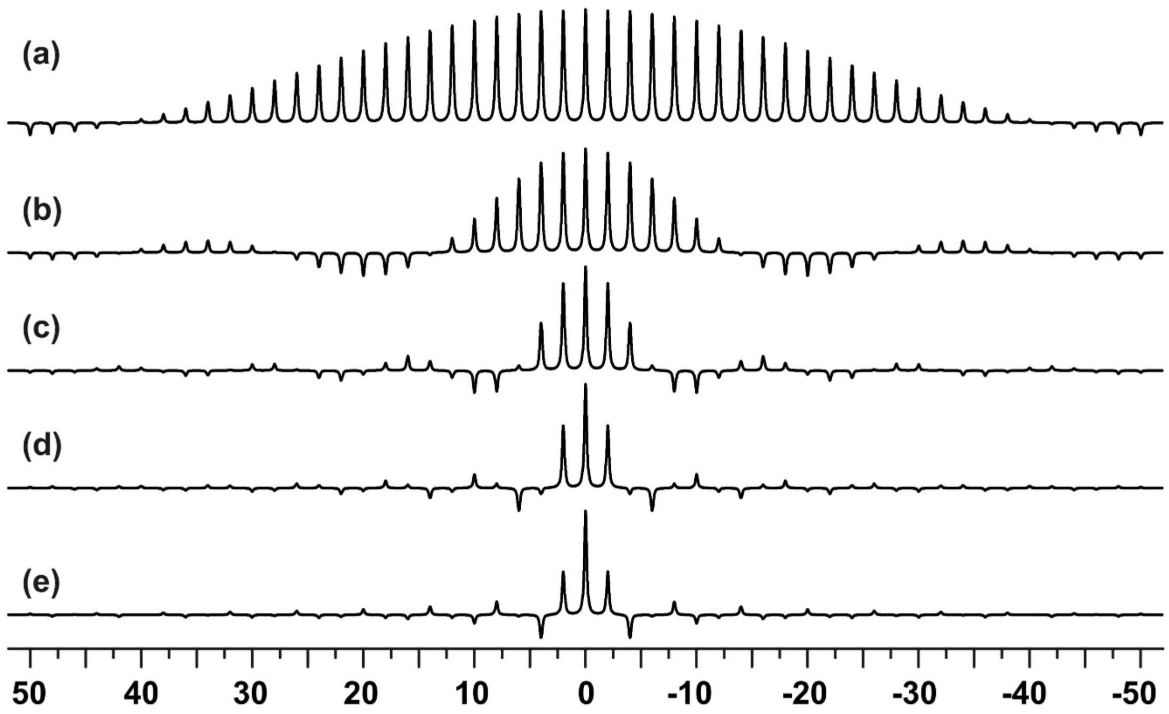

Figure S2. Excitation profiles for GEMSTONE simulated using Spinach [see reference 16 in the main text]with different durations, τp, (a)-(e) of 10, 25, 50, 80, and 100 ms for the adiabatic pulses. An 18.5 ms Rsnob (100 Hz effective bandwidth) pulse was used for the selective 180° pulse, giving uniform refocusing over the range of offsets mapped. A spin system containing 51 isolated protons was used, allowing a single calculation to be used for each profile. As expected, the selectivity improves in inverse proportion to τ_p. No excitation sidebands are seen, in contrast to CSSF (see Figure S3a).

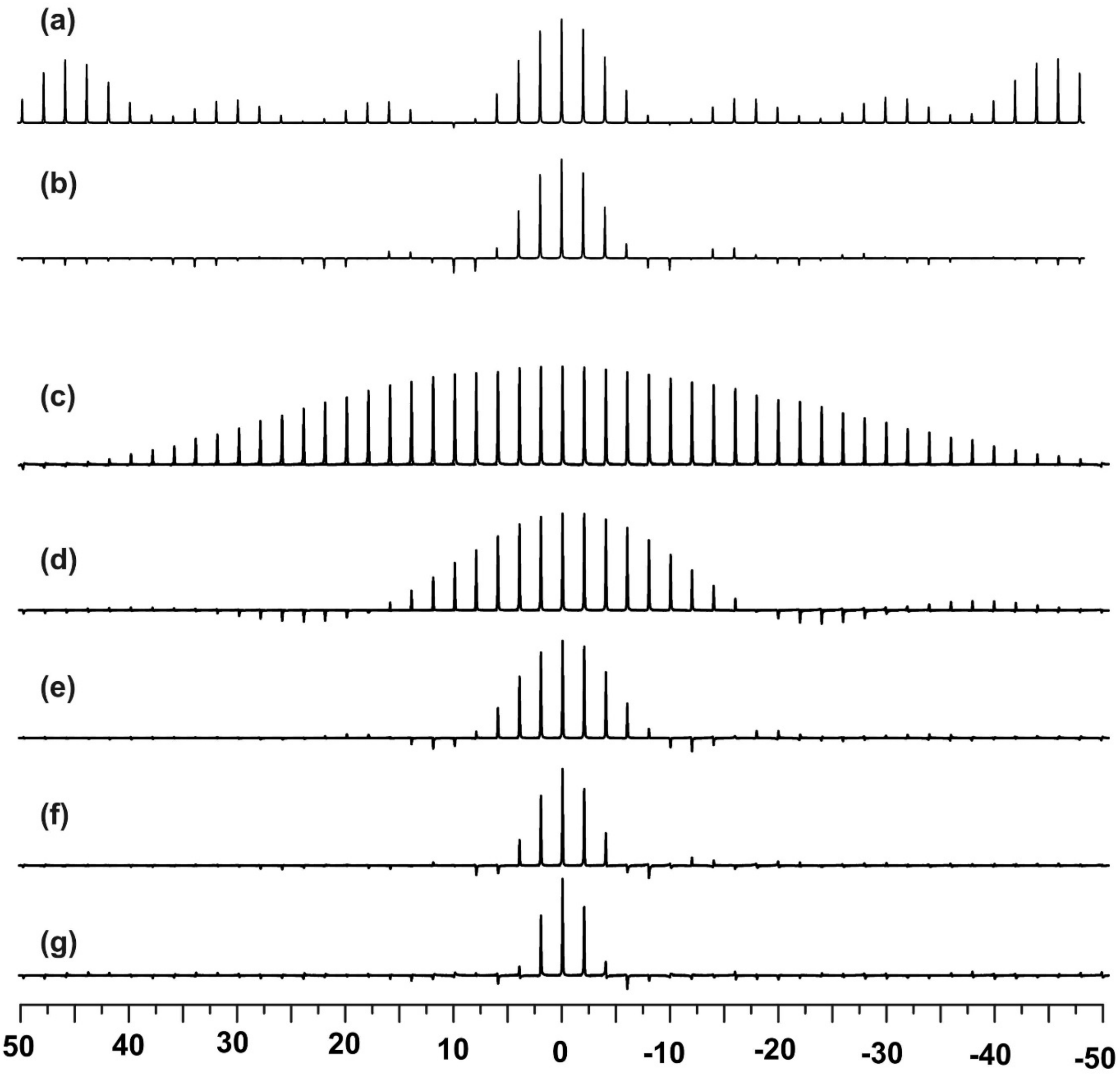

Figure S3. Excitation profiles mapped using a doped water sample with (a-b) conventional VT-CSSF as implemented in TopSpin 4.0.7, and (c-g) the single-scan GEMSTONE experiment. The transmitter offset was changed in steps of 2 Hz over ±50 Hz from resonance; the chemical shift evolution time t1max was set to 80 ms, and 4 and 16 increments were used in (a) and (b) respectively. The GEMSTONE experiment used five different durations τp for the adiabatic pulses (c)-(g): 10, 25, 50, 80, and 100 ms, τp. The VT-CSSF excitation profile is, as expected, complicated both by relaxation and, in (a), where the selectivity of the 180° pulse was insufficient, by the presence of multiple excitation sidebands caused by the limited number of t_1 increments used. This complicates direct comparison of the first null in the CSSF and GEMSTONE profiles, but they both have the expected general *sinc* shape. The conventional 180° selective pulse was 18.5 ms Rsnob (100 Hz effective bandwidth) in all experiments, giving uniform refocusing over the range of offsets mapped. Total experiment times were (a) 26, (b) 111, and each of (c-g) 4 min respectively.

3. Example: 17β-estradiol

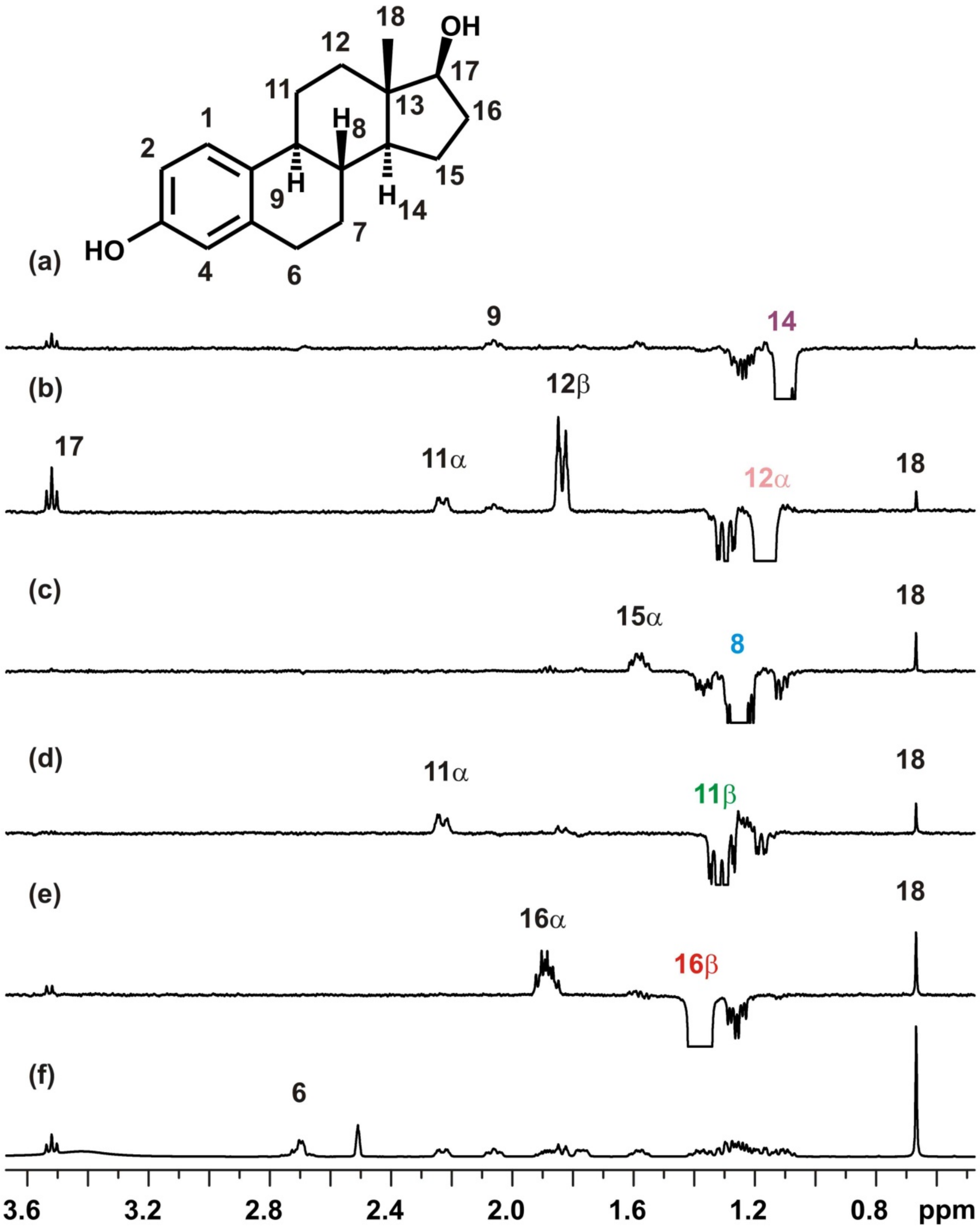

Figure S4. (a)-(e) GEMSTONE NOESY experiments and (f) ^{1}H NMR spectra of estradiol in DMSO-d_6. Conventional selective 1D NOESY experiments are not applicable to this example because of severe multiplet overlap in the region between 1.0 and 1.4 ppm.

4. Example: Carbocycle 1

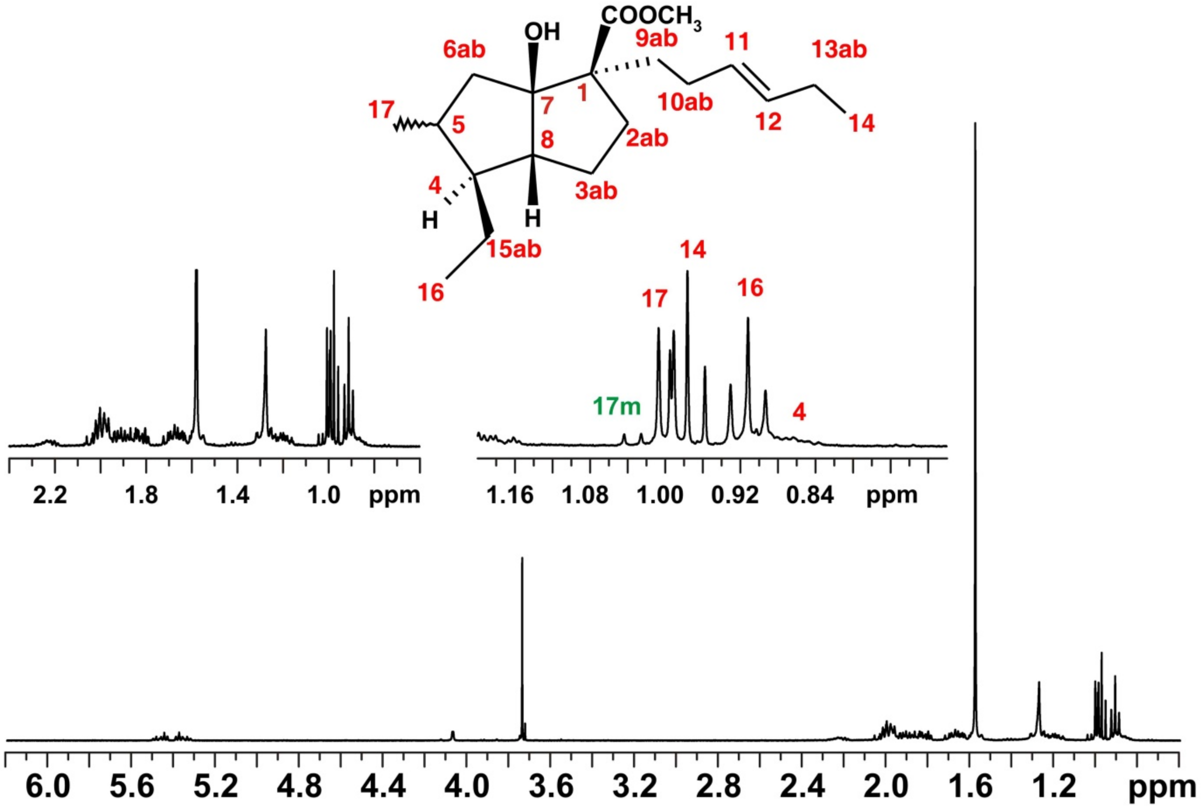

Figure S5. ^{1}H NMR spectrum of carbocycle **1** in $CDCl_3$. The sample contains major and minor diastereomers which differ in chirality at position 5.

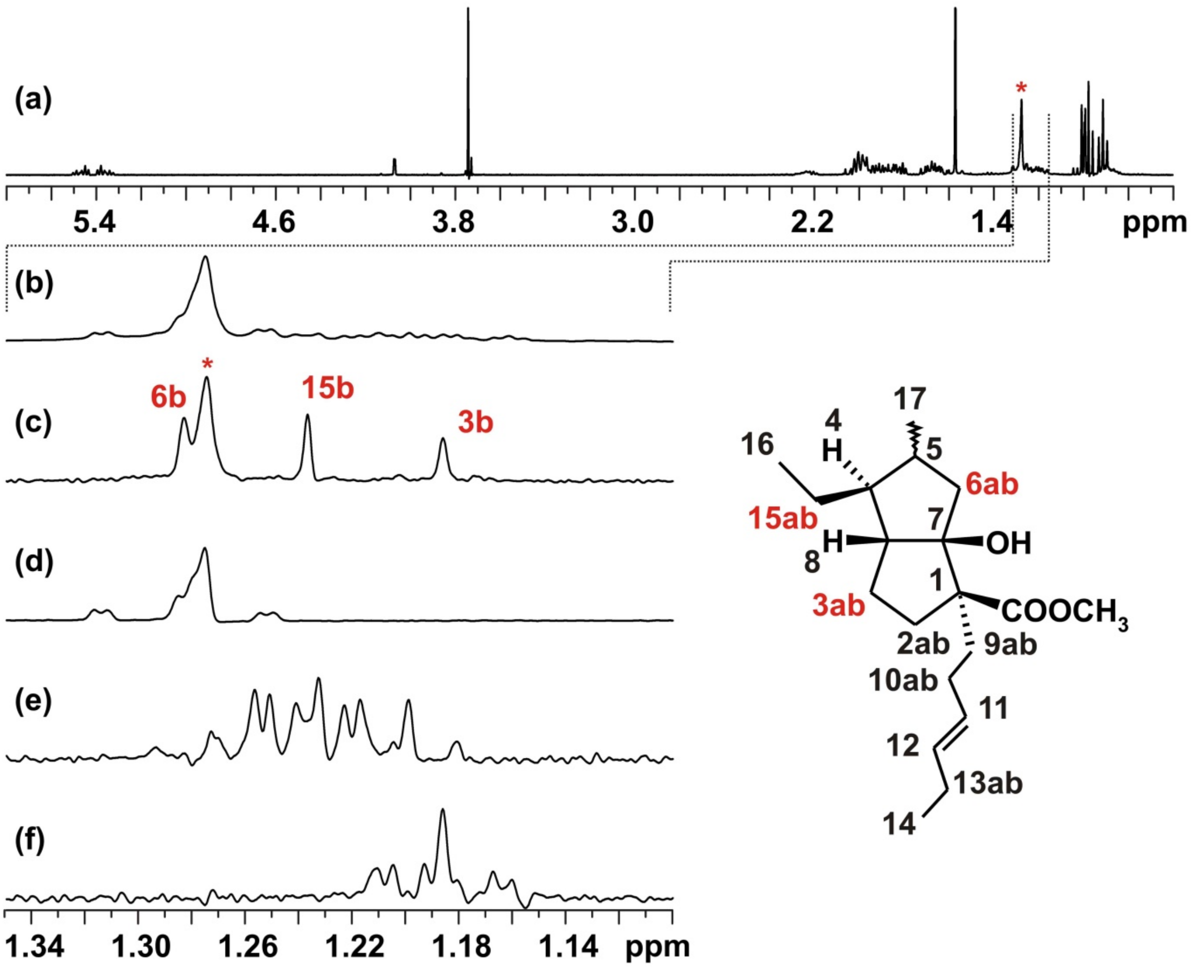

Figure S6. (a) ^{1}H NMR spectrum of carbocycle **1** in $CDCl_3$ and (b-f) expansion of overlapped region. (b) Conventional ^{1}H NMR spectrum, (c) band-selective pure shift NMR spectrum, (d-f) GEMSTONE selective excitation of the three proton multiplets in this region. The singlet marked by an asterisk is from a structurally unrelated impurity. Conventional selective excitation of the three overlapping multiplets in this region is not possible.

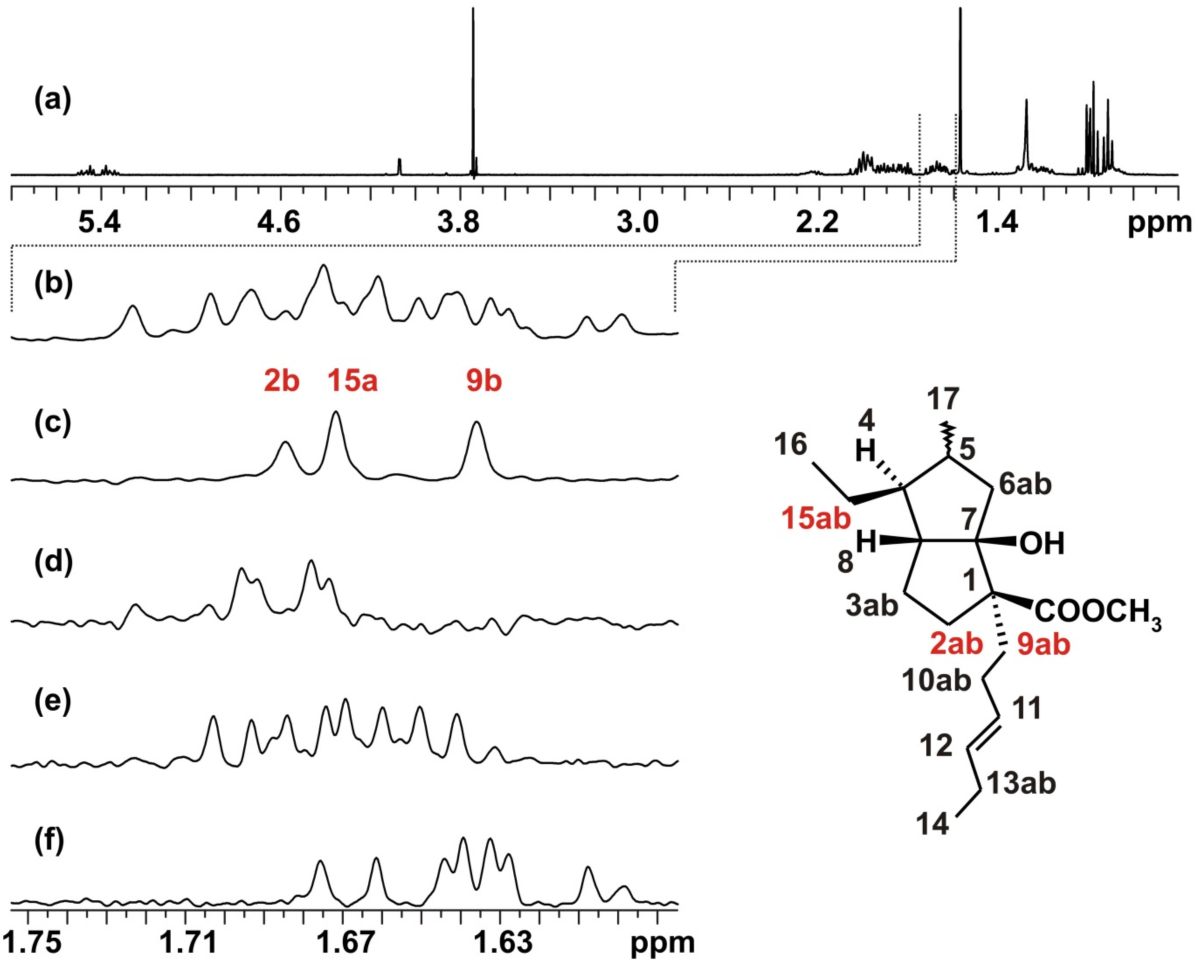

Figure S7. (a) ^{1}H NMR spectrum of carbocycle **1** in $CDCl_3$ and (b-f) expansion of the overlapped region. (b) Conventional 1H NMR spectrum, (c) band-selective pure shift NMR spectrum, (d-f) GEMSTONE selective excitation of the three proton multiplets in this region. Conventional selective excitation of each of the overlapping multiplets in this region is not possible.

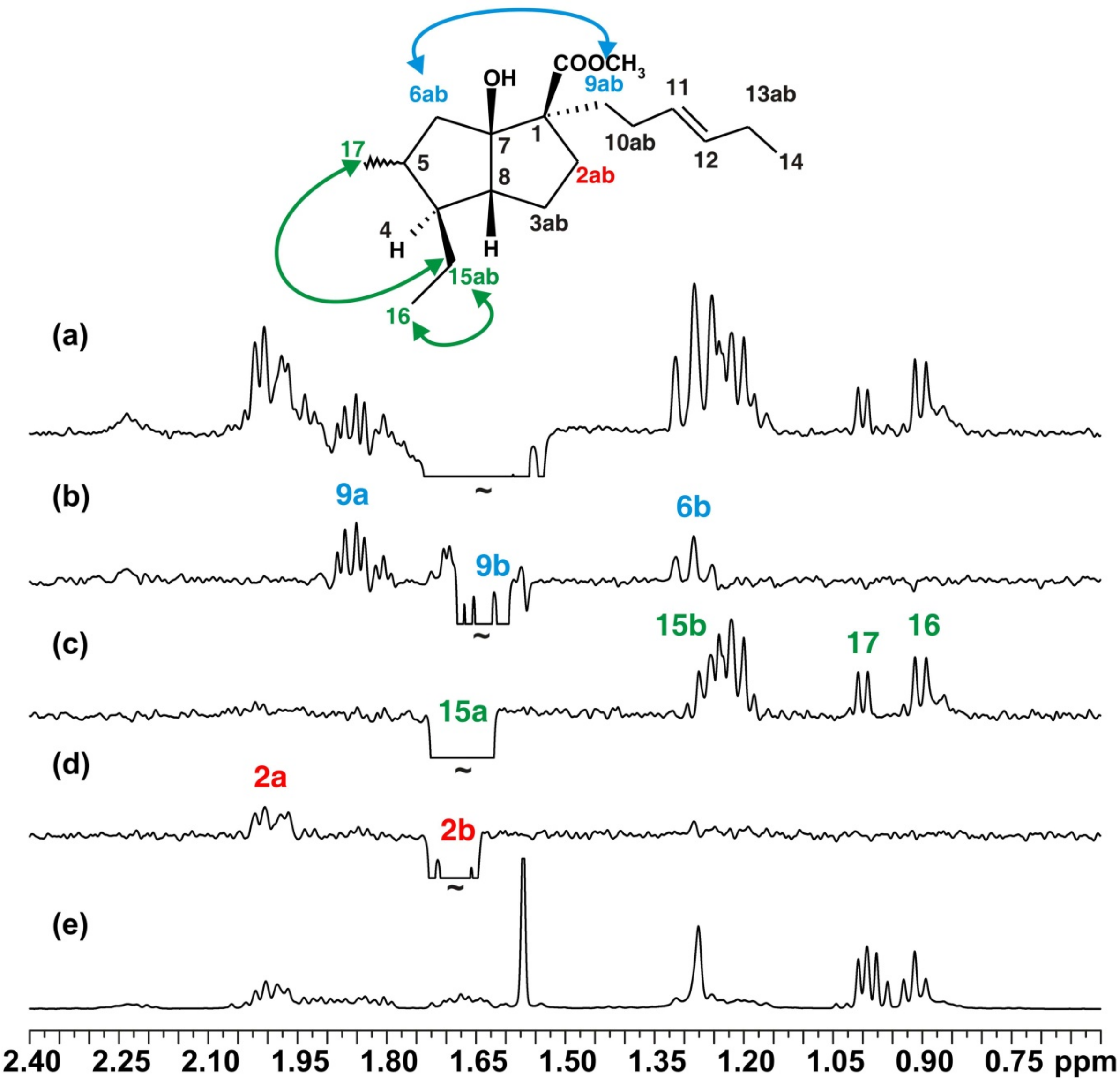

Figure S8. (a) Conventional selective 1D NOESY, and (b-d) 1D GEMSTONE NOESY spectra of carbocycle **1** in $CDCl_3$. Conventional selective excitation provides ambiguous NOEs, but the individual NOE contacts are clearly distinguished in the GEMSTONE NOESY spectra.

5. Example: Carbocycle 2

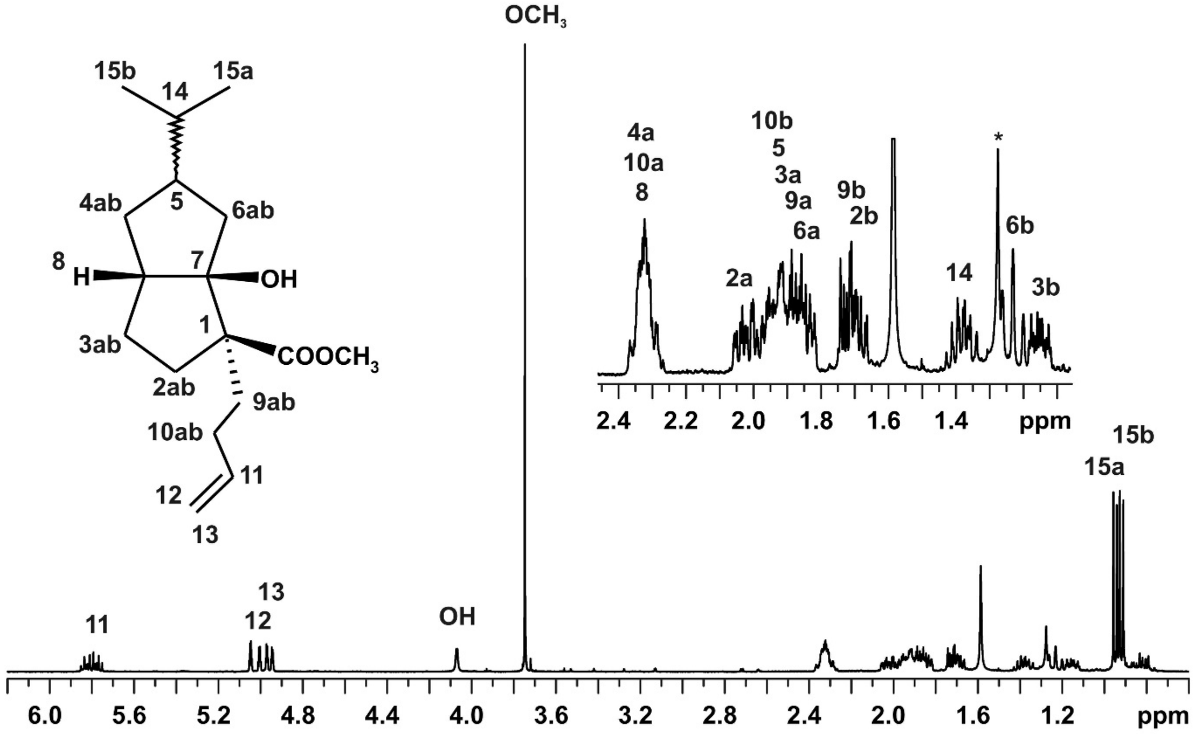

Figure S9. ^{1}H NMR spectrum of carbocycle **2** in $CDCl_3$. The sample contains mainly one stereoisomer, for which the configuration at position 5 was established using the GEMSTONE 1D NOESY experiments reported here.

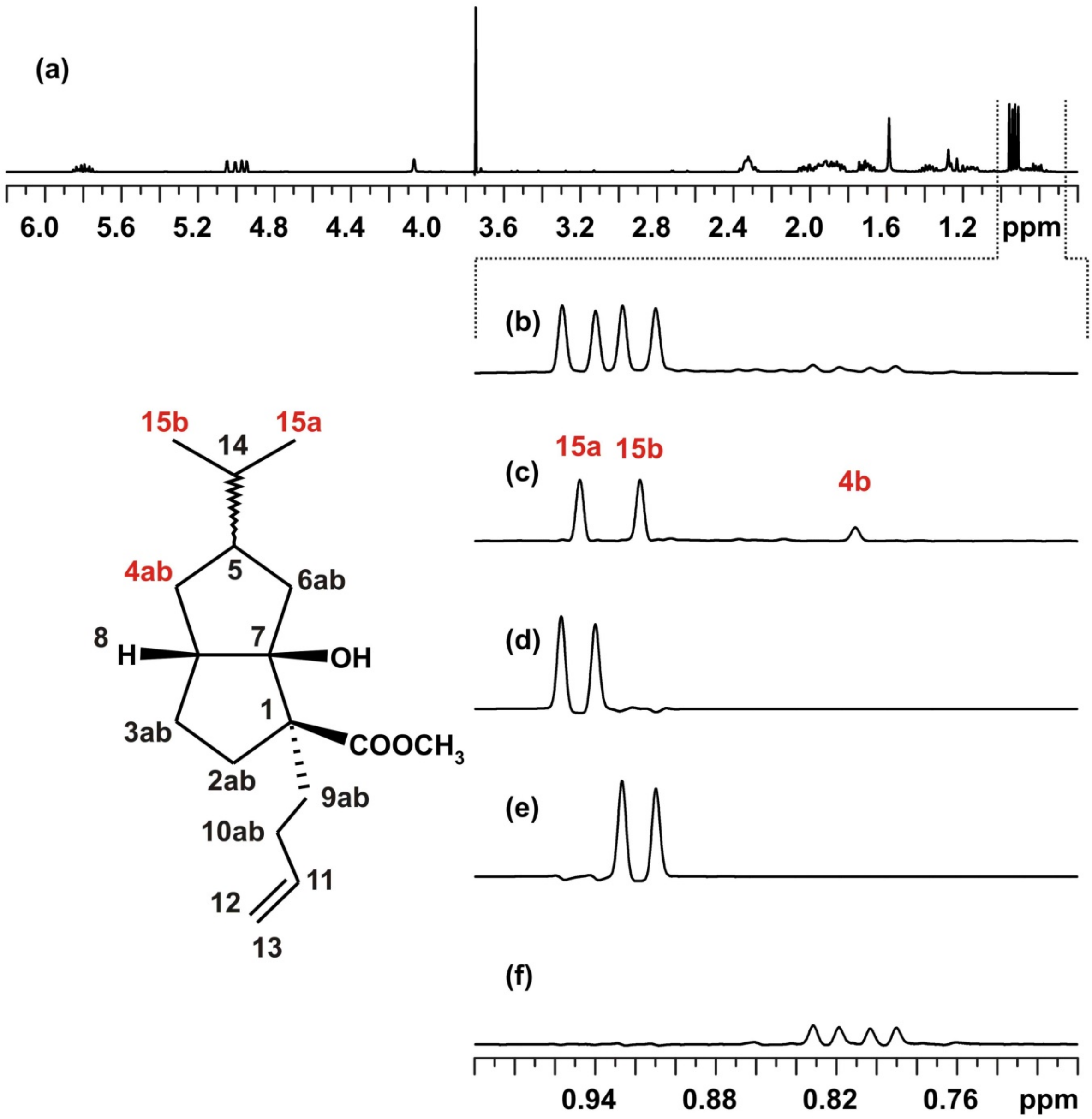

Figure S10. (a) ^{1}H NMR spectrum of carbocycle **2** in CDCl3 and (b-f) expansion of overlapped region. (b) Conventional ^{1}H NMR spectrum, (c) Zangger-Sterk pure shift NMR spectrum, (d-f) GEMSTONE selective excitation of the three proton multiplets in this region.

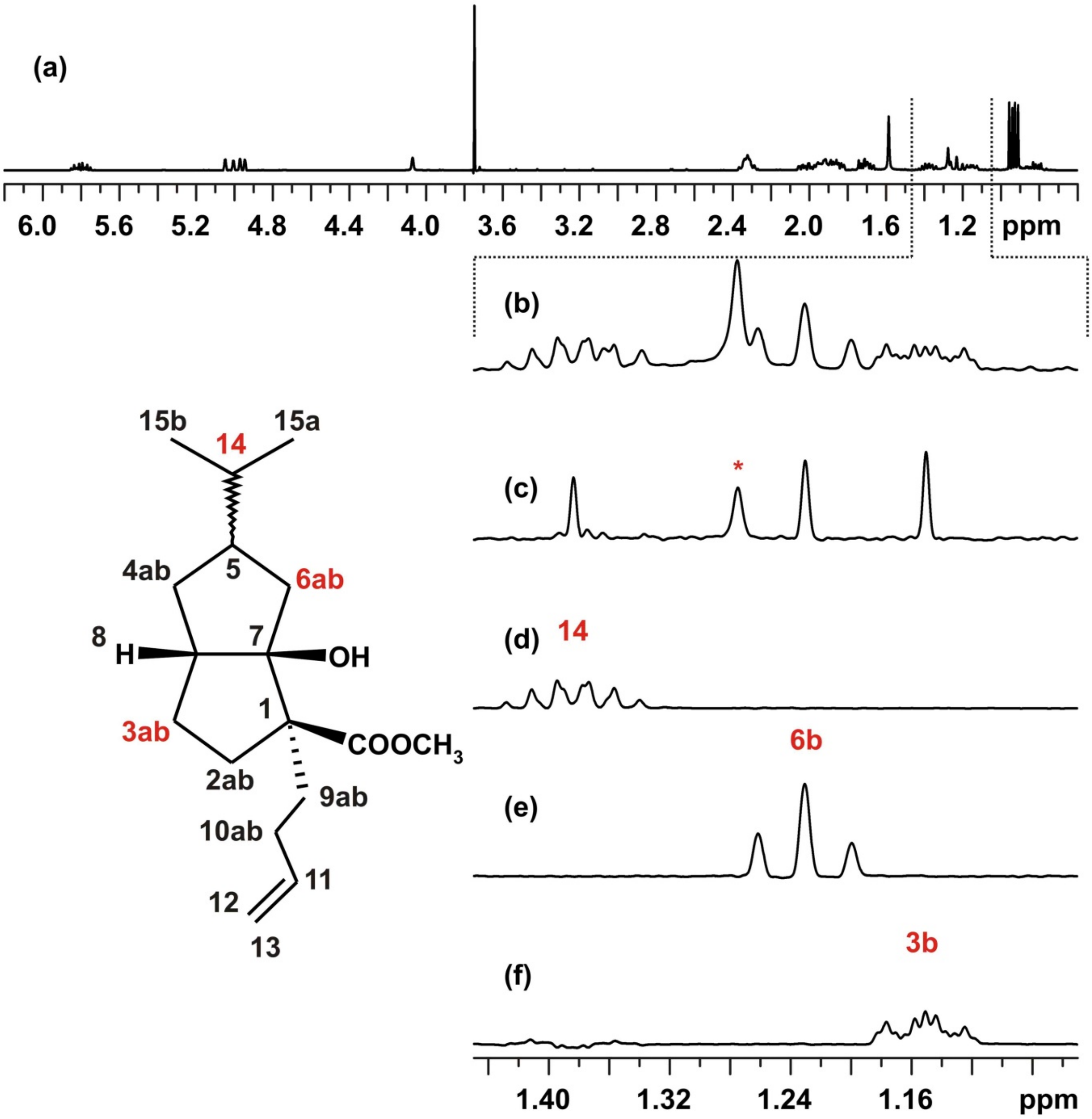

Figure S11. (a) ^{1}H NMR spectrum of carbocycle **2** in $CDCl_3$ and (b-f) expansion of overlapped region. (b) Conventional ^{1}H NMR spectrum, (c) Zangger-Sterk pure shift NMR spectrum, (d-f) GEMSTONE selective excitation of the three proton multiplets in this region. The singlet marked by an asterisk is from a structurally unrelated impurity.

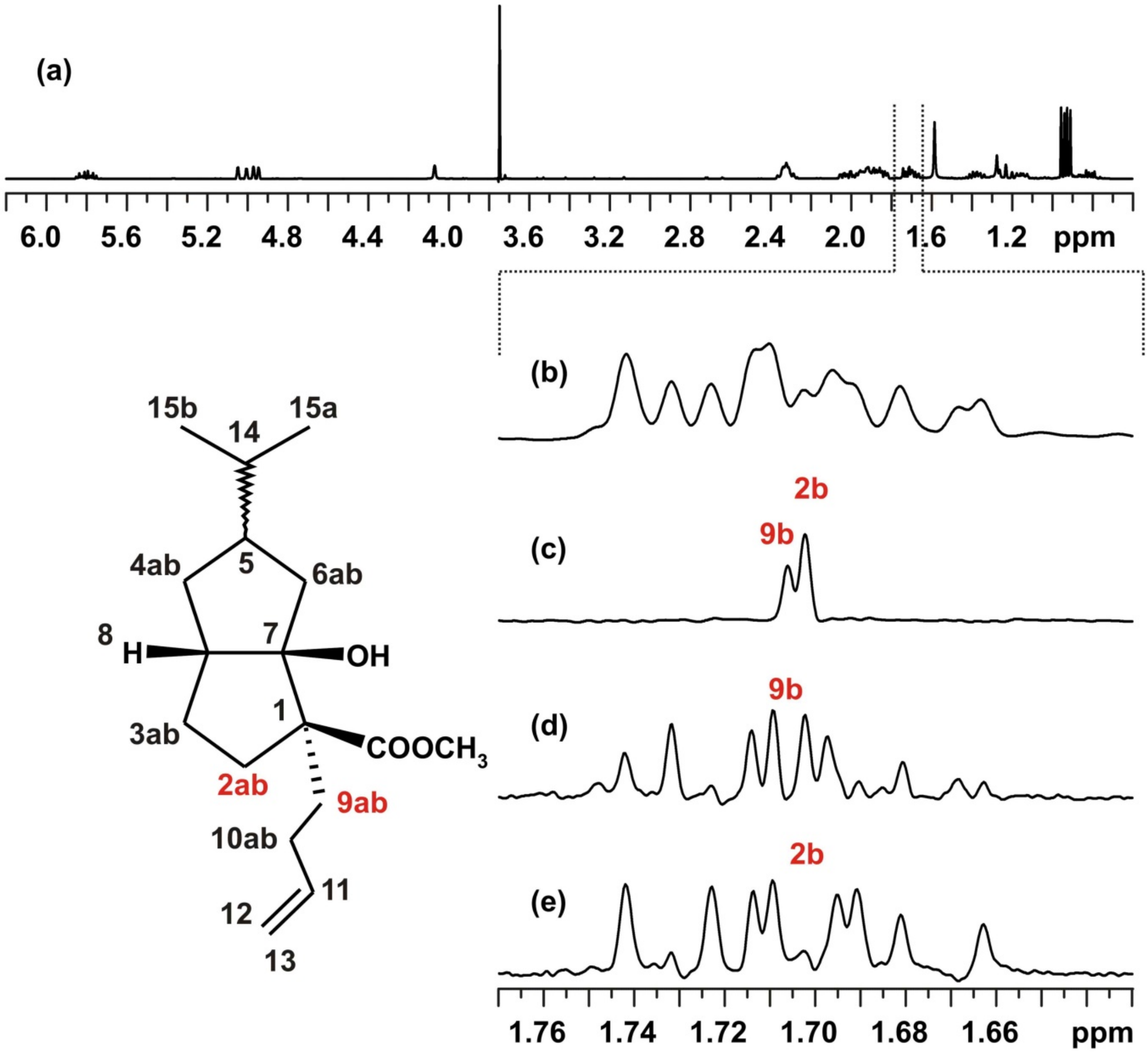

Figure S12. (a) ^{1}H NMR spectrum of carbocycle **2** in $CDCl_3$ and (b-e) expansion of a severely overlapped region. (b) Conventional ^{1}H NMR spectrum, (c) band-selective pure shift NMR spectrum, (d-e) GEMSTONE selective observation of the two significantly overlapped proton multiplets. Conventional selective excitation for this example is not possible; even for GEMSTONE this is a very difficult example. Perfect selectivity is not achieved but the two multiplet structures can be sufficiently disentangled in the GEMSTONE spectra. It can be seen in the selective spectra that some of the multiplet lines overlap exactly while others do not. The apparent asymmetry in the multiplets is caused by strong coupling' the roofing effect is more significant for 9b. Success in selectively exciting these multiplets is critical to its application in 1D GEMSTONE NOE experiments (see Figure S15).

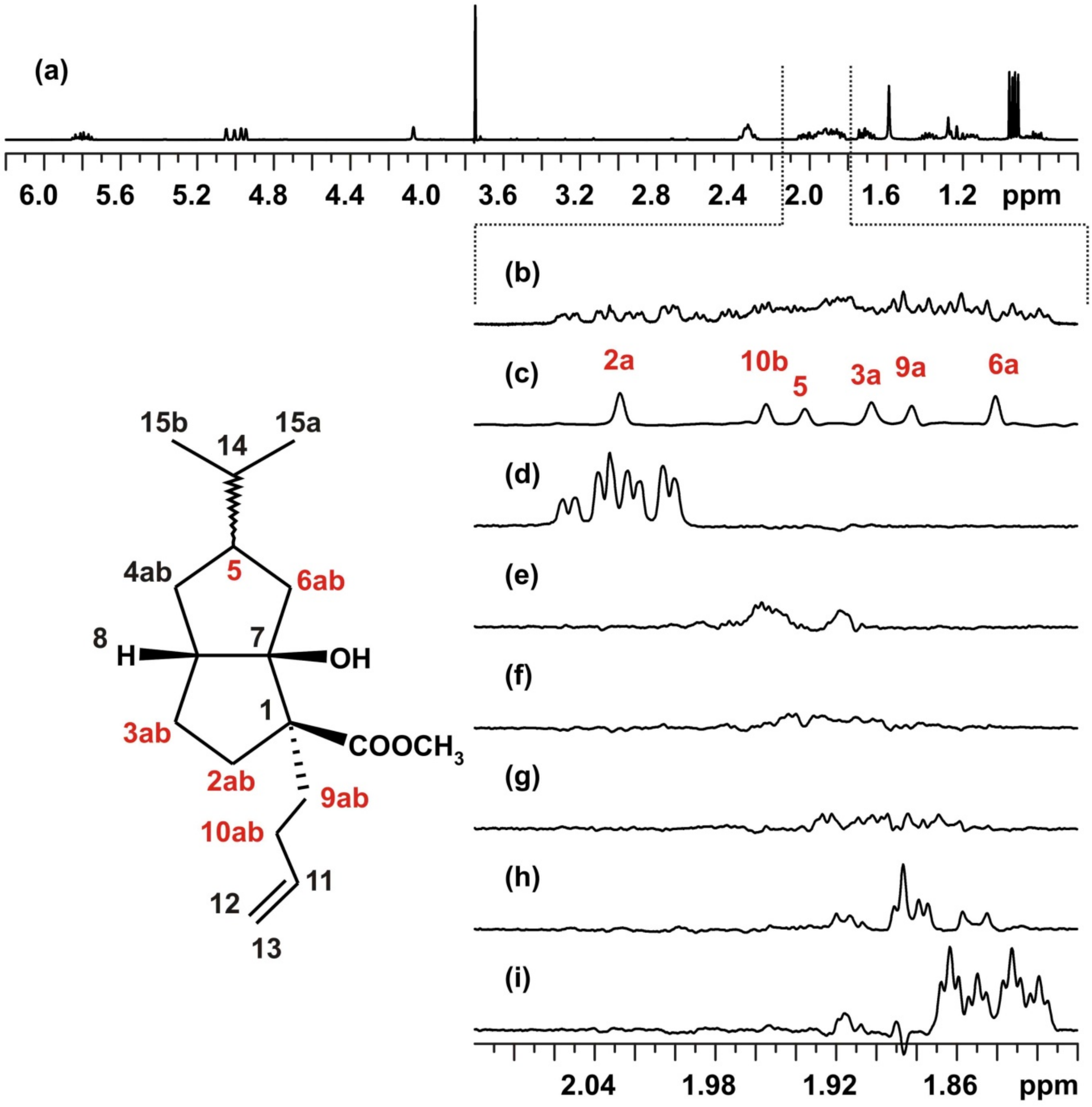

Figure S13. (a) ^{1}H NMR spectrum of carbocycle **2** in $CDCl_3$ and (b-f) expansion of an overlapped region. (b) Conventional ^{1}H NMR spectrum, (c) Zangger-Sterk pure shift NMR spectrum, (d-i) GEMSTONE selective observation of the proton multiplets in this region. Conventional selective excitation of the overlapping multiplets in this region is not possible. Strong coupling limits the practical use of GEMSTONE experiment in some cases.

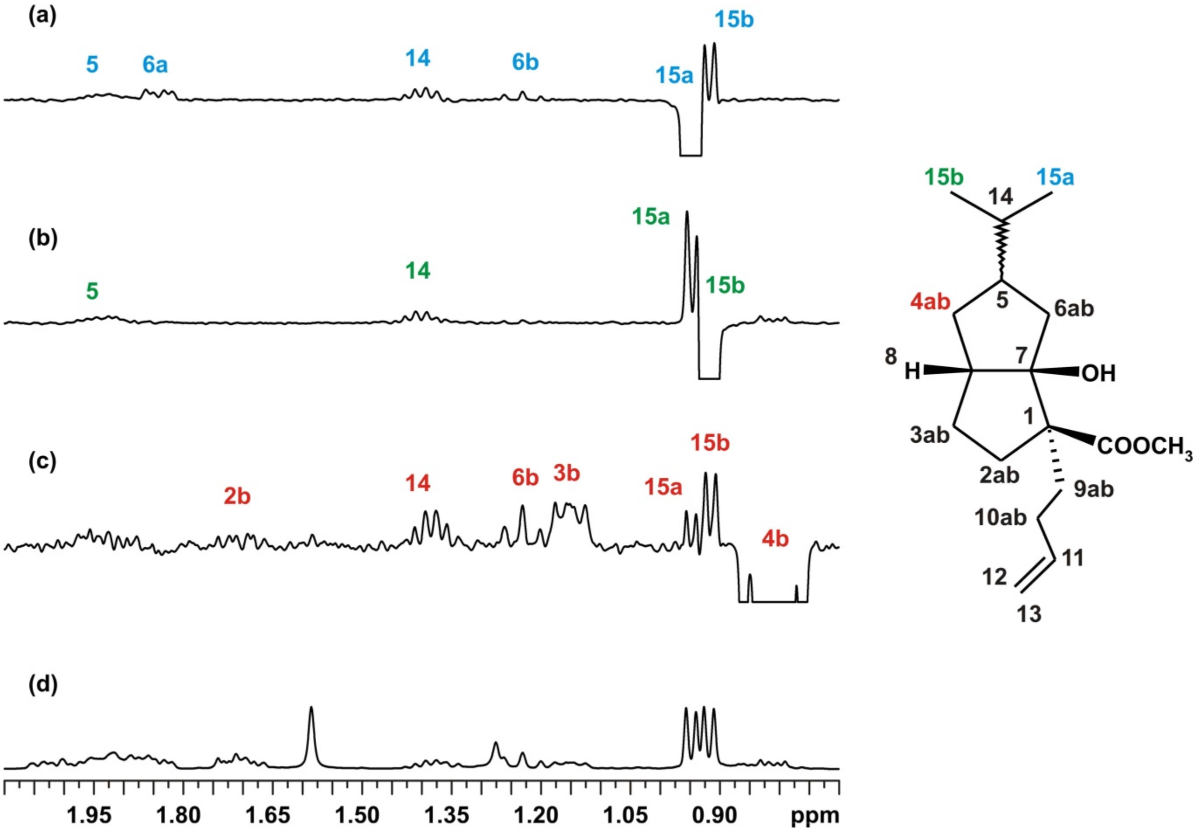

Figure S14. (a-c) 1D GEMSTONE NOESY and (d) ^{1}H NMR spectra of carbocycle **2** in $CDCl_3$. The positive signals observed in (a-b) for 15a and 15b are not caused by insufficient selectivity, but are the expected trivial NOEs in the isopropyl group. The selectivity of the GEMSTONE method without NOE is shown in Figure S10.

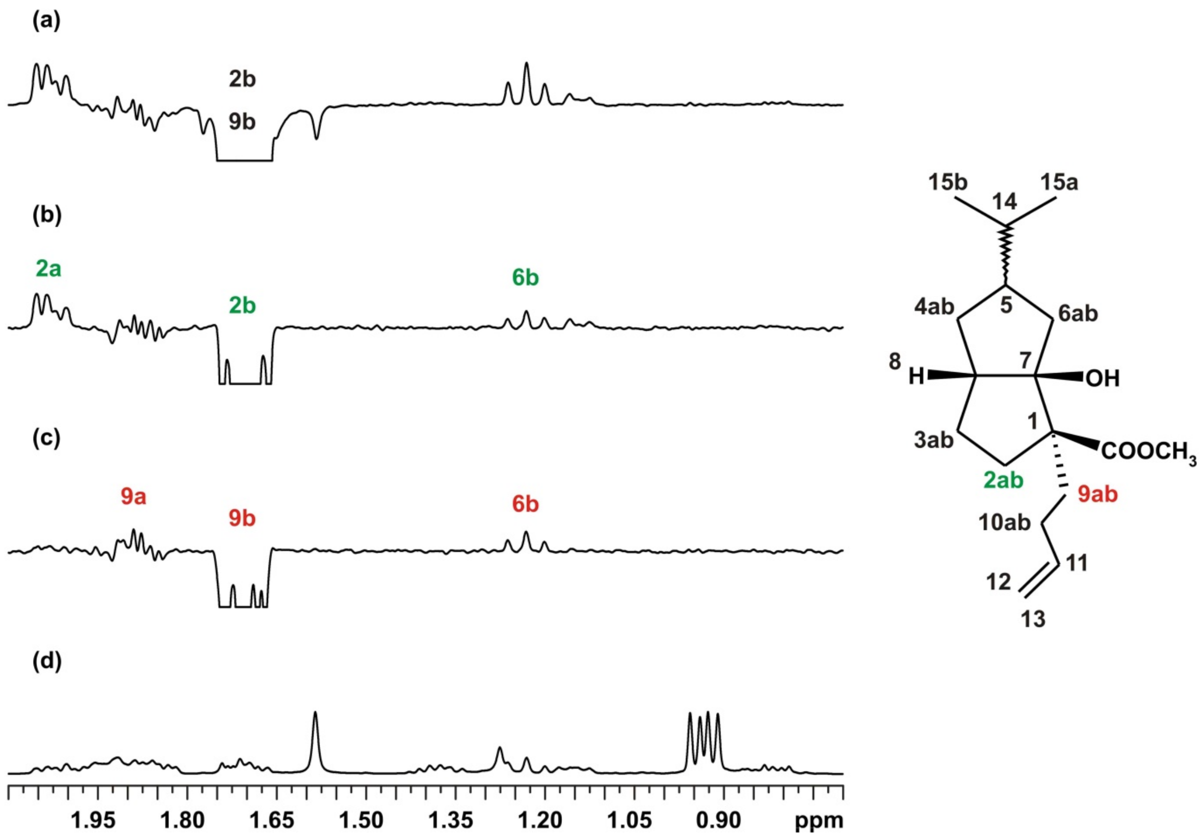

Figure S15. (a) Conventional selective 1D NOESY, (b-c) 1D GEMSTONE NOESY and (d) ^{1}H NMR spectra of carbocycle **2** in $CDCl_3$. Conventional selective excitation gives ambiguous NOEs, but GEMSTONE separates 2b and 9b NOEs cleanly. See the selective excitation of the multiplets and the extent of overlap in Figure S12.

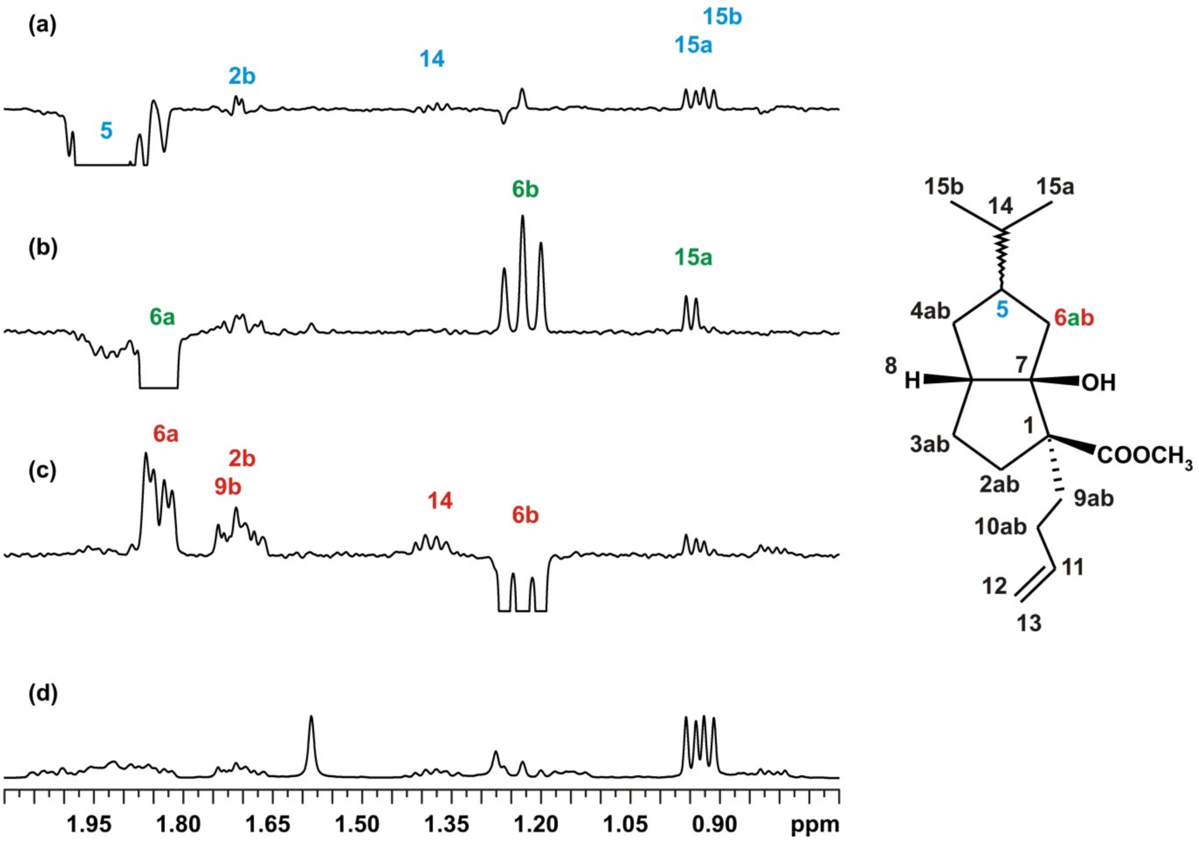

Figure S16. (a-c) 1D GEMSTONE NOESY and (d) ^{1}H NMR spectra of carbocycle **2** in $CDCl_3$. Conventional selective excitation of proton 5 is impossible because it overlaps with other protons. The NOE observed between 5 and 2b is indicative of the stereochemistry.

6. Example: mixture of cinchona alkaloids

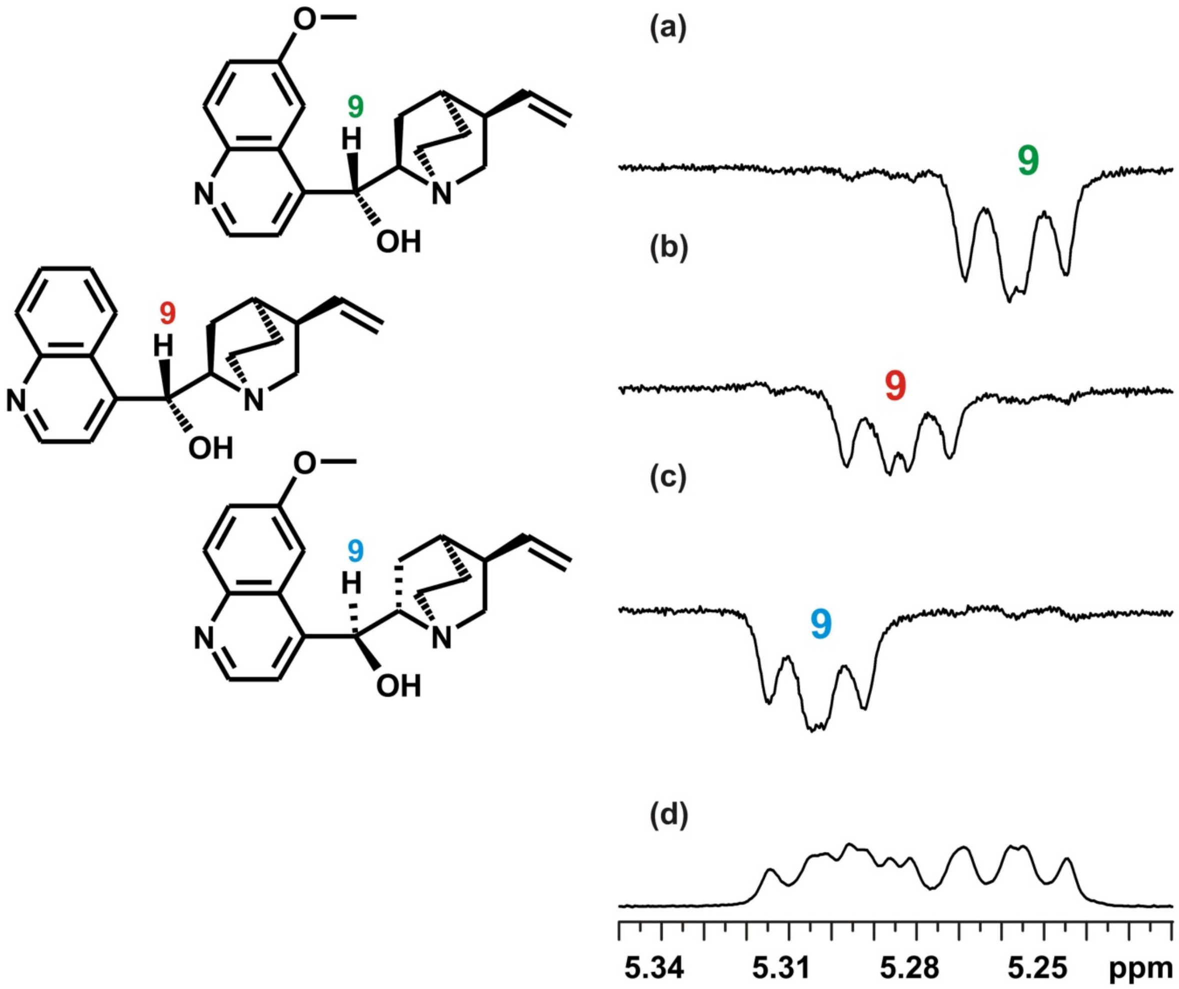

Figure S17. Part of (a-c) 1D GEMSTONE NOESY using zero mixing time and (d) ^{1}H NMR spectra of cinchona alkaloid mixture in DMSO-d_6. Clean selection of the multiplets of proton 9 from each component is shown.

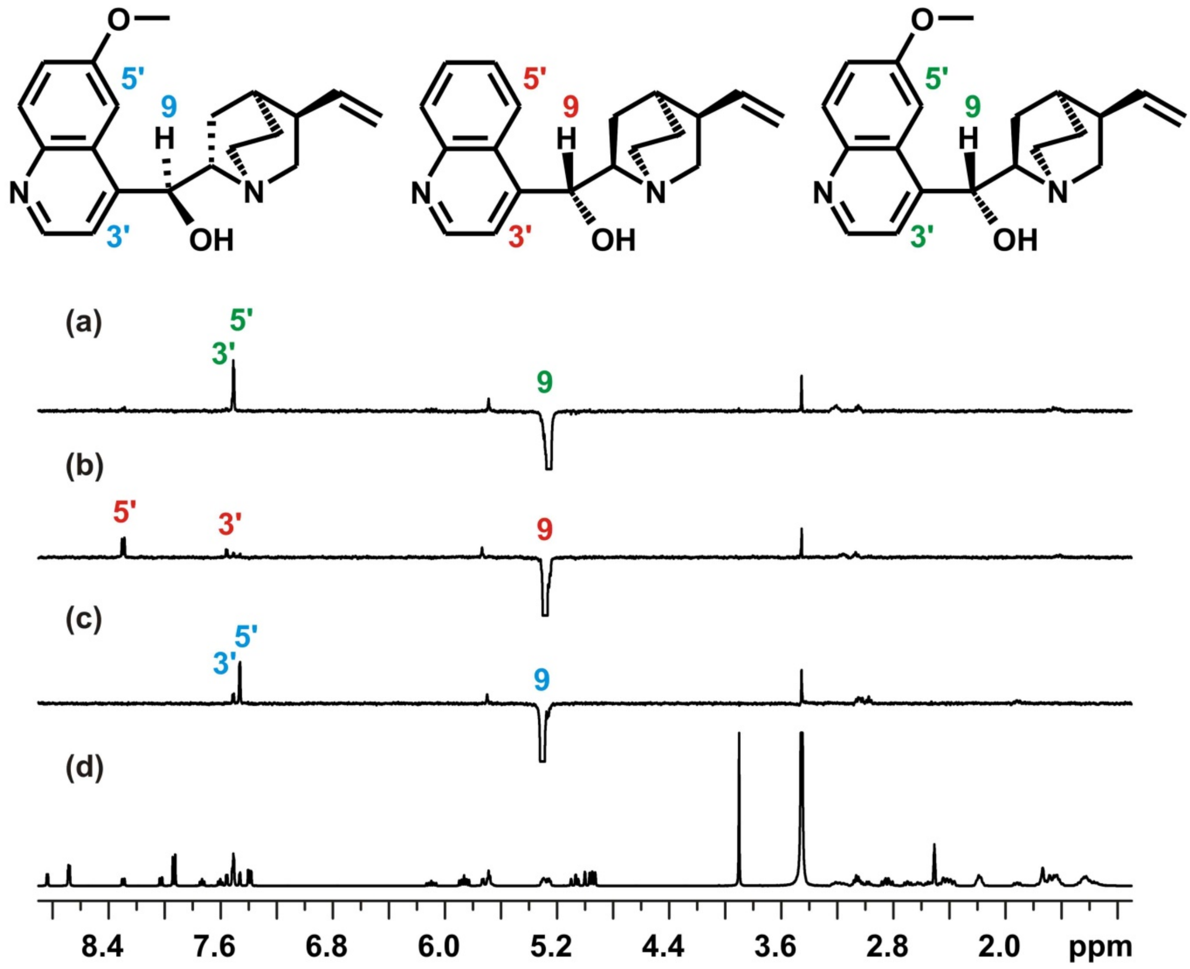

Figure S18. (a-c) 1D GEMSTONE NOESY and (d) ^{1}H NMR spectra of cinchona alkaloid mixture in DMSO-d_6. Clean selection of the different multiplets of proton 9 is shown in Figure S17.

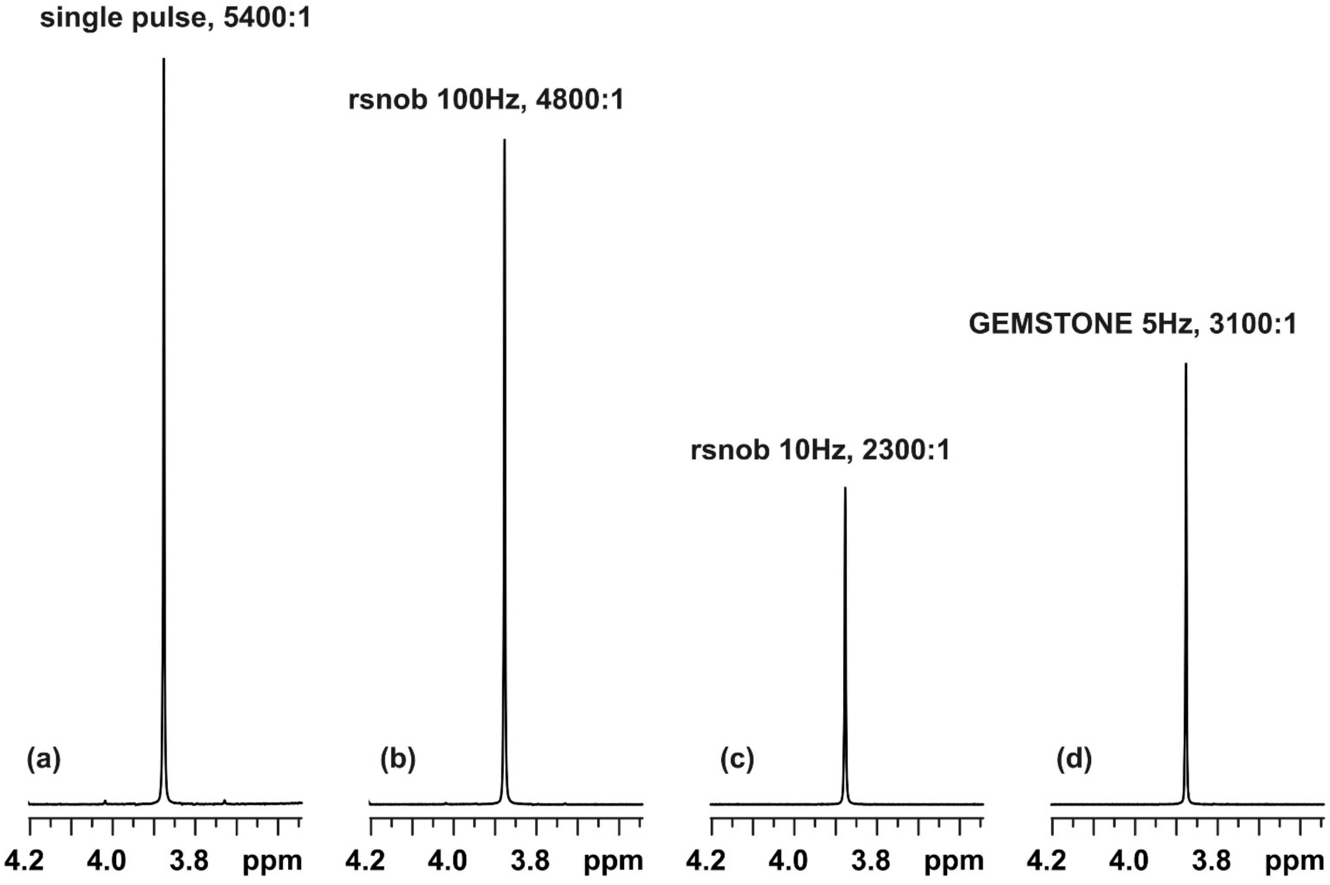

Figure S19. Comparison of (a) single pulse acquire and (b-d) selective excitation of the well-resolved methoxy resonance in the cinchona alkaloid mixture in DMSO-d_6 for (b) SPFGE using an 18.5 ms RSNOB pulse of 100 Hz bandwidth, (c) SPFGE using a 185 ms RSNOB pulse of 10 Hz bandwidth, and (d) GEMSTONE using τ_p=100 ms, which gives a half-height bandwidth of 5 Hz. The signal-to-noise ratios of the spectra, which were acquired using a single scan, are respectively 5400, 4800, 2300, and 3100:1. Comparison between (b) and (c) shows the sensitivity reduction for increased selectivity in SPFGE; comparison between (c) and (d) shows that GEMSTONE is competitive with, and can give better sensitivity than, SPFGE. Half-height bandwidths of 10 Hz for RSNOB and 5 Hz for GEMSTONE were chosen to give a fair comparison, as RSNOB has a better excitation profile than the basic GEMSTONE method (see Fig. S3g). The relative sensitivities of the different experiments are a complex function of bandwidth, relaxation characteristics, pulsed field gradient parameters and diffusion coefficients. Here the penalty due to relaxation losses is greater in (d) the GEMSTONE experiment than in (c), where conventional excitation is used but with twice the bandwidth, but conventional selective excitation suffers more from diffusional signal attenuation caused by the field gradient pulses needed either side of the long selective pulse. In GEMSTONE the equivalent source of diffusion losses can be kept much smaller because the selective pulse is only used to refocus J-modulation, the selectivity being provided by the adiabatic pulses. In GEMSTONE the bandwidth of the adiabatic pulses is typically more than an order of magnitude smaller than that used in applications such as zero-quantum suppression or ultrafast NMR; coupled with a correspondingly weak gradient, this keeps diffusion losses low.

7. Author Contributions

PK, RWA, NK and MN conceived the project. PK implemented and designed the project, performed the experiments, and wrote the first draft. NK, MPP and DJP provided the materials for the two carbocycle examples. PK, RWA, MN and GAM wrote the final manuscript. GAM, MN and RWA supervised the project; the first also refined the GEMSTONE pulse sequence design during the project. All authors read and approved the final manuscript.
